# Older People With Type 2 Diabetes—Individualizing Management With a Specialized (OPTIMISE) Community Team: Protocol for a Safety and Feasibility Mixed Methods Study

**DOI:** 10.2196/13986

**Published:** 2019-06-07

**Authors:** Rajna Ogrin, Sandra Neoh, Tracy Aylen, Ralph Audehm, Leonid Churilov, Lorenna Thurgood, Georgia Major, Jeffrey Zajac, Elif I Ekinci

**Affiliations:** 1 Bolton Clarke Research Institute Bolton Clarke Bentleigh Australia; 2 Austin Health Clinical School University of Melbourne Heidelberg Australia; 3 Austin Health Heidelberg Australia; 4 Bolton Clarke Forest Hill Australia; 5 Department of General Practice University of Melbourne Parkville Australia; 6 Florey Institute of Neuroscience and Mental Health University of Melbourne Heidelberg Australia; 7 RMIT Unversity Melbourne Australia; 8 National Ageing Research Institute Parkville Australia; 9 Department of Endocrinology Austin Health Heidelberg Australia

**Keywords:** type 2 diabetes mellitus, aged, person-centered, telehealth

## Abstract

**Background:**

The prevalence of diabetes is rising in older people. In 2018, over 574,000 Australians reported having diabetes. The highest prevalence (19.4%) of diabetes has been observed in people aged 85 years and older. Clinical guidelines recommend that diabetes management should be individualized; however, there is limited information regarding the current management patterns of diabetes in older people, given most clinical trials exclude participants from this age group. Available data identify that few individuals achieve optimal glycemic levels in the general population, potentially leading to adverse health outcomes and impact on quality of life. The data on glycemic profiles of older population are limited.

**Objective:**

The aim of this study is to examine individualized diabetes management intervention for older people through home visits with a credentialed diabetes educator (CDE) and telehealth consultations with an endocrinologist located at a tertiary hospital.

**Methods:**

This paper describes the design and methodology of a mixed methods feasibility and safety study to identify the current management of type 2 diabetes in people aged 65 years or older. We will implement and evaluate a personalized approach to management in the community of an Australian metropolitan city. This management approach will utilize flash glucose monitoring and home visits with the support of a community home nursing service CDE and telehealth consultation with an endocrinologist located at a local tertiary hospital.

**Results:**

The study commenced in February 2017 and has recruited 43 participants, with final data collection to be completed by July 2019. Data analysis will commence after final data collection, with results expected to be published by the end of 2019.

**Conclusions:**

This study is the first of its kind to explore individualized diabetes management for community-dwelling older people, with an aim to achieve optimal glycemic levels (glycated hemoglobin between 53 and 69 mmol/mol [7%-8.5%] depending on the fitness and frailness of the older individual). The data drawn from this study may be used to inform policy makers, service providers, clinicians, and older adults living with diabetes.

**International Registered Report Identifier (IRRID):**

DERR1-10.2196/13986

## Introduction

The prevalence of type 2 diabetes in older Australians is increasing. In 2015, 1 in 6 Australians aged 65 years or older reported living with diabetes [1]; the incidence of this disease peaks between the ages of 65 and 74 years [2]. Diabetes care is particularly challenging in older adults because of the longer duration of the disease and the increased likelihood of complications, comorbidities, falls, and polypharmacy [3]. In addition, there is a lack of an evidence base regarding the safety and efficacy of diabetes pharmacotherapy in this population.

International guidelines recommend that diabetes care and glycemic targets should be individualized with consideration of the patient’s age, comorbidities, functional status, and living situation [4,5]. In particular, treatment of hyperglycemia should be carefully balanced with the avoidance or minimization of hypoglycemia [6]. This is often difficult in older individuals with long durations of diabetes and possible underlying secondary pancreatic failure.

Currently in Australia, health care delivery of diabetes management in older adults is conducted through primary care or hospital-based diabetes clinics. These environments are not necessarily ideal, as they do not easily allow assessments of home situations and true functional status [7]. An alternative approach is telemedicine, defined by the American Telemedicine Association as the use of medical information exchanged from one site to another via electronic communications to improve a patient’s clinical health status. Telemedicine has been successfully used for the management of chronic diseases in this age group [8]. The current approach involves the credentialed diabetes educator (CDE) using their laptop to link into Web videoconferencing in the home of the person living with diabetes to link with the endocrinologist located in the hospital for joint initial and final review appointments. This approach has been used effectively previously, with good acceptability [9].

We hypothesize that a specialist-led telemedicine service, including flash glucose monitoring technology, is a safe and feasible method of delivering diabetes care to older Australians in Melbourne, Victoria. This study is the first of its kind to trial individualized diabetes management plans for older people through home visits with a CDE and telehealth consultations with an endocrinologist located at a tertiary hospital [10].

## Methods

This protocol paper structure is per the Standard Protocol Items for Clinical Trials guidelines [11].

### Study Design and Setting

This is a feasibility and safety trial, using Simon’s 2-stage design [12], of a new model of health care. This study will use a mixed methods approach describing quantitative glycemic data and qualitative interviews regarding person-centered outcomes.

The study will include older individuals, aged 65 years or older, living with type 2 diabetes, and residing in the northern region of metropolitan Melbourne. The Older People with Type 2 diabetes—Individualizing Management wIth SpecializEd (OPTIMISE) team delivering care consists of the following: (1) a CDE working with a home service organization providing home visits and (2) an endocrinologist based at the tertiary teaching hospital. Joint telehealth consultations will be conducted with the endocrinologist at the hospital and the CDE with participants with diabetes in the participants’ home, including the use of flash glucose monitoring data collected by participants in the preceding 2 weeks to inform clinical decision making.

### Aim

The primary aim is to trial the safety and feasibility of a new model of diabetes care, aimed to optimize diabetes management and improve quality of life at home using a specialized OPTIMISE community team.

The secondary aims are as follows:

1. To describe the current diabetes management
2. To trial the effectiveness of a specialized community-based team to optimize diabetes management and improve quality of life of older people with type 2 diabetes.

### Primary Research Questions

The primary research questions are as follows:

1. Among community-dwelling older adults with type 2 diabetes, is the OPTIMISE model of care safe?
2. Among community-dwelling older adults with type 2 diabetes, is the OPTIMISE model of care feasible?

The OPTIMISE model will be deemed safe if all the following conditions are met:

1. There are no deaths associated with diabetes intervention.
2. There are no serious adverse events causally related to the intervention as adjudicated by a panel of physicians within 1 week of the event.
3. The participant spends <30% of time in severe hyperglycemia (>20 mmol/L) measured by flash glucose monitoring, and
4. The participant spends <5% of time in hypoglycemia (<4 mmol/L) measured by flash glucose monitoring.

The OPTIMISE model will be deemed as feasible if the following conditions are met:

1. The approached participant agrees to proceed with the intervention at the first visit by the CDE.
2. The participant completes the 4-month intervention by supplying a flash glucose monitor data capture rate of at least 40% (defined as the number of 15-min time points with glucose values captured divided by the total number of 15-min time points for the duration the sensor was applied); 40% was chosen as the feasibility value as previous larger clinical trials involving younger participants with type 1 and 2 diabetes have accepted 50% as clinically sufficient data [13,14].
3. The participant attends at least 80% of scheduled follow-up appointments.

### Secondary Research Questions

The secondary research questions were as follows:

1. What are the perceptions of older adults with type 2 diabetes regarding their experiences of telehealth consultations in delivering home-based diabetes care?
2. What are the perceptions of older adults with type 2 diabetes regarding using flash glucose monitoring technology to support diabetes management?
3. What are the perceptions of the OPTIMISE team care providers regarding their experiences with this model of care?

The outline of the participant pathway for this study is shown in Multimedia Appendix 1.

### Participants

There will be 2 groups of participants:

1. Older adults with type 2 diabetes.
2. Health care providers within the OPTIMISE team.

#### Older Adults With Type 2 Diabetes

Potential participants will be eligible for the study if they are aged 65 years or older, have been diagnosed with type 2 diabetes, can speak and understand English, and live in the community nursing organization’s catchment area for home visits.

Individuals will be excluded from the study if they

- Are unable to consent
- Are unable to self-manage their diabetes or do not have a caregiver to provide support
- Are already under the care of an endocrinologist
- Have an acute condition that destabilizes their glycemic levels or are likely to require hospital readmission
- Are in residential care or palliative care

#### Health Care Providers in the Older People With Type 2 Diabetes Individualizing Management With Specialized Team

The endocrinologist and CDE involved in the study will also be invited to participate in the research.

### Recruitment

Participants with type 2 diabetes will be recruited from several sources, which are as follows:

1. Existing home nursing clients: the CDE will screen and identify eligible participants from the Northern site of the home nursing organization.
2. Tertiary hospital: the endocrinologist will screen new referrals to the diabetes clinic.
3. Tertiary hospital: the endocrinologist will screen all recently admitted inpatients with glycated hemoglobin (HbA_1c_) level of greater than or equal to 6.5%.
4. Referrals from other clinicians in the region: diabetes educators, geriatricians, and general practitioners who have working relationships with the research team will be invited to refer eligible participants.
5. Self-referrals from the community who meet eligibility criteria.

Once identified, the CDE will contact potential participants to inform them of the study. If interested, the first home visit will be arranged to explain the study and answer questions. If the participant agrees to proceed, a second home visit is organized to discuss the participant information and consent form and obtain informed consent.

Health care providers involved in the OPTIMISE team will be invited to participate, provided with the participant information and consent form, and given the opportunity to ask questions.

### Baseline Clinical Data Assessments

Once recruited, the CDE will organize a third home visit with the participant for baseline data collection; the details are shown in Table 1.

**Table 1:** Older people with type 2 diabetes individualizing management with specialized baseline participant and biochemistry data collection.

Baseline participant information components	Content of the baseline participant information
Demographic data	Age, gender, country of birth, language(s) spoken, education level, and socioeconomic index for areas
Descriptive data	Height, weight, blood pressure, medical history, medical diagnoses, medication names, doses and directions of use, and history of falls in the past 12 months
Mini Nutritional Assessment-Short Form [15]	Nutrition
Rowland Universal Dementia Assessment Scale [16]	Dementia or Cognitive Impairment: The Rowland Universal Dementia Assessment Scale
Charlson Comorbidity Index [17]	Prognostic comorbidity for mortality
Diabetes-specific information	Duration of diabetes, family history, current pharmacotherapy, relevant comorbidities, hypoglycemia, and hyperglycemia risk
Biochemistry data collection	Glycated hemoglobin, Liver function tests, lipid profile, fasting plasma glucose, renal function, full blood examination, and spot urinary albumin to creatinine ratio

### Intervention

The intervention involves the implementation of a management plan developed after a telehealth consultation with the endocrinologist at the hospital linking with the CDE and participant with diabetes in the participant’s home. The management plan is based on the clinical assessment and the profile of data collected using the flash glucose monitor in the preceding 2 weeks. The participant with diabetes is then supported by the CDE to implement the management plan, through face-to-face visits and phone calls. A final follow-up telehealth consultation with the endocrinologist at the hospital, linking with the CDE and participant with diabetes in the participant’s home is undertaken. In this consultation, an ongoing plan for diabetes management is decided based on assessment outcomes, including the profile of data collected using the flash glucose monitor in the preceding 2 weeks. Specific details on each component are provided below.

#### Flash Glucose Monitoring

During the third home visit, the flash glucose monitoring system (Freestyle Libre, Abbott Diabetes Care Inc) will be placed on the back of the participant’s upper arms by the CDE, as per the manufacturer’s guidelines. The system involves a very small glucose-sensing filament (<0.4 mm thick and 5 mm long) worn under the skin and connected to a water-resistant, plastic on-body patch the size of a 1 dollar coin. The participant is shown how to scan the sensor using the supplied touchscreen reader device and asked to scan the sensor at least 4 times per day for 2 weeks and advised how to interpret the results and act on them.

#### Biochemistry Collection

The participant will be contacted by a research officer to organize a home visit to complete quantitative questionnaires and to arrange biochemistry collection (Table 1).

#### Telehealth Consultation With Participant, Credentialed Diabetes Educator, and Endocrinologist

The fourth home visit (week 2, Multimedia Appendix 1) by the CDE occurs 2 weeks after the flash glucose sensor is applied. Data from the flash sensor are downloaded and emailed to the endocrinologist for review. The CDE and participant then teleconference with the endocrinologist, located at the tertiary hospital, using the Skype software (Microsoft Corporation, Luxembourg) installed on the CDE’s laptop. An individualized care plan will be developed, which may involve the initiation or adjustment of oral medicine, injectable therapy initiation or adjustment, and diabetes education.

The management plan will be underpinned by goal-setting theory, where the process of goal setting facilitates behavior change by guiding people’s effort and attention [18]. Feedback strategies will be incorporated into the goal-setting practices to enhance goal attainment [19].

Treatment care plans are based on current best practice guidelines [3,20]. Education will be individually tailored to meet the needs of the participant [3,20]. The endocrinologist will inform the participant’s general practitioner of the care plan and recommendations via a letter.

#### Care Plan Implementation and Monitoring

The CDE will implement the care plan with participants and will facilitate referrals to local community services where required. The CDE will contact the participants monthly for the occurrence of adverse events and will report this accordingly. Additional telehealth consultations with or without repeat flash glucose monitoring with the endocrinologist will be scheduled only if changes to management plans are required. Participants may discontinue the intervention at any time.

### Follow-Up Assessments

At week 18, the CDE will visit the participant to apply the flash glucose monitor sensor, collect weight and blood pressure data, apply the flash glucose monitor sensor, and provide a repeat pathology slip. At week 20, the CDE will return to the participant’s home, download the flash glucose data, and email this to the endocrinologist, just before a repeat telehealth consultation, using Skype.

At this consultation, the participant, endocrinologist, and CDE will determine if further diabetes support is required. Either ongoing diabetes care will be handed back to the general practitioner via a letter or a referral will be made to the diabetes clinic at the tertiary hospital if ongoing specialist endocrine support is required.

After the final telehealth consultation, the research officer will arrange a final home visit for quantitative data collection and a semistructured qualitative interview. The interview will be audio-taped and transcribed verbatim. The interview will start with an open question asking the participants to describe their experiences of the care they received from the team. Prompts will be used, if needed, to ensure inclusion of the following pivotal information: their perceptions of being seen by the CDE in their home; the experience of seeing the specialist over the computer, including the performance of telehealth such as connectivity; their experiences of using the flash glucose monitor; what they thought could have been improved, and what worked well in the care delivery; how confident they were in managing their diabetes; if they sought outside help for their diabetes management; and whether they would recommend this program to others with diabetes.

A pathology slip for a follow-up final HbA_1c_ is emailed to the participant to be performed at week 32.

At the end of the trial, the CDE and endocrinologist will be invited to participate in a face-to-face interview, which will be audio-taped and transcribed, to ascertain their experiences of providing care to participants. The interview will start with an open question asking them to describe their experiences of the care they provided as part of the team. Prompts will be used, if needed, to ensure inclusion of the following pivotal information: their satisfaction with their role in the team; their experiences of working with the endocrinologist or CDE; their experiences of using the flash glucose monitor; the experience of using telehealth, including the performance of telehealth such as connectivity; whether they encountered safety concerns during the study; what are their thoughts about the impact of this program on the participants with diabetes; what they thought could have been improved and what worked well in the care delivery; and if the program were to be run as business as usual at their organization, whether they would want to be a part of the team.

The intervention will be deemed as both feasible and safe, worthy of further investigation in a subsequent randomized comparative trial, if it proceeds to the second phase of Simon’s 2-stage design [12] and more than 31 out of 43 participants do not experience feasibility or safety issues.

To evaluate the impact of the program on participants, secondary outcome measures will be collected, including: biochemical markers, person-centered measures, and service data (shown in Multimedia Appendix 2 [21-25]).

### Data Management

The research officer will manage the study data and enter data into a statistical database, supervised by the project leads. No names will be used on any data collection forms, and all data will be deidentified when entered into the database. Electronic databases will be kept on secure, password-protected drives on a secure network, and hard copy data will be stored in a locked cupboard in a secure area. Data entry will be undertaken by research officers involved in the study, with random 10% of data checked for accuracy. Range checks for data values will be undertaken to ensure any errors are identified.

### Sample Size

Simon’s 2-stage design is used where a group of participants is enrolled in the first stage, and depending on the successful outcome of this group, a second group of participants is then enrolled [12]. In the first stage, 11 participants will be recruited. If 7 or more participants do not experience feasibility or safety issues, the study will proceed to phase 2, where 32 additional participants are recruited for a total of 43. The study design yields a type 1 error rate of 0.05 and power of 0.8 when the true proportion of patients without a negative composite feasibility/safety outcome is 0.8.

### Statistical Methods/Analysis

Baseline clinical, social, demographic, and biochemical data will be presented as counts (proportions), means (SDs), or medians (interquartile ranges), depending on the nature of the underlying distribution. The proportions of patients with specific outcomes will be reported with corresponding 95% CIs.

If more than 31 out of 43 participants do not experience feasibility or safety issues, the intervention will be deemed worthy of further investigation in a subsequent randomized comparative trial.

Statistical analysis will be undertaken by STATA (ICv 14) software (StataCorp, College Station, TX).

Interviews with participants will be analyzed thematically using a constant comparative approach [26]. Transcripts will be read by researchers, emergent themes discussed, and interpretations compared.

### Safety Considerations and Dissemination

The project has ethical oversight from the organizational human research ethics committee (HREC): home nursing organization HREC (HREC, project number 183) and from the tertiary hospital HREC (project HREC/16/Austin/496). In the case of major adverse events, both HRECs will be informed on the same day by the project manager. Confidentiality and security of participant data will involve deidentification and the use of codes generated using a random number generator. The principal investigators will conduct auditing of the dataset at the end of data entry, following data analysis to ensure compliance with the protocol. Access to data will only be given to researchers involved in the trial. Investigators will publish results of findings in peer-reviewed journals and present the findings at conferences associated with diabetes management and health services research. There are no publication restrictions.

## Results

The project was funded in November 2016 and approved by the respective organizations (as noted above) in February 2017. Data collection commenced in July 2017, with anticipated data collection to cease in July 2019; 43 participants have consented to participate, with data analysis to commence after final data collection is complete. We anticipate results to be published by the end of 2019.

## Discussion

This study is the first of its kind to trial the safety and feasibility of a new model of diabetes care, aiming to optimize diabetes management and improve quality of life in the home using a specialized community team involving CDE home visits and telehealth consultations with an endocrinologist.

The increasing prevalence of type 2 diabetes, particularly in older adults, highlights the importance of identifying knowledge gaps and potential risks in the management and care of older people living with this disease. This study is unique in trialing person-centered home-based diabetes care, which adheres to current guidelines and recommendations [3]. We will describe the current management and interventions implemented in this cohort, as well as the key issues and perceptions of the older community members receiving the care. The perceptions of the providers on delivering this care will also be explored.

The strength of this project is its ability to comprehensively assess the feasibility and safety of this multifaceted home-based model of care. This study also uses new technology such as telehealth and flash glucose monitoring and will add to the growing evidence base for use of technology in supporting diabetes self-management in older adults.

Potential limitations of this study include its small sample size and lack of participants from non–English-speaking backgrounds. Furthermore, the study may be prone to ascertainment bias because of convenience sampling from a geographic location limited to 1 community nursing organization and 1 tertiary hospital.

We anticipate that the data generated by this project will be used to inform service providers, clinicians, and older adults living with diabetes of the care currently provided, how guidelines for optimal treatment translate into practice, and to inform future research of the effectiveness of this kind of multimodal intervention.

## Appendix

Multimedia Appendix 1 Flowchart of participant pathway.

Multimedia Appendix 2 Study outcome measures and data collection.

## Abbreviations

CDE: credentialed diabetes educator
HREC: human research ethics committee
OPTIMISE: Older People with Type 2 diabetes—Individualizing Management wIth SpecializEd
