# Supplementary figures and images for: Older People With Type 2 Diabetes—Individualizing Management With a Specialized (OPTIMISE) Community Team: Protocol for a Safety and Feasibility Mixed Methods Study
Source: JMIR Res Protoc. 2019 Jun 7;8(6):e13986. doi: 10.2196/13986 (PMC6592394; doi:10.2196/13986)

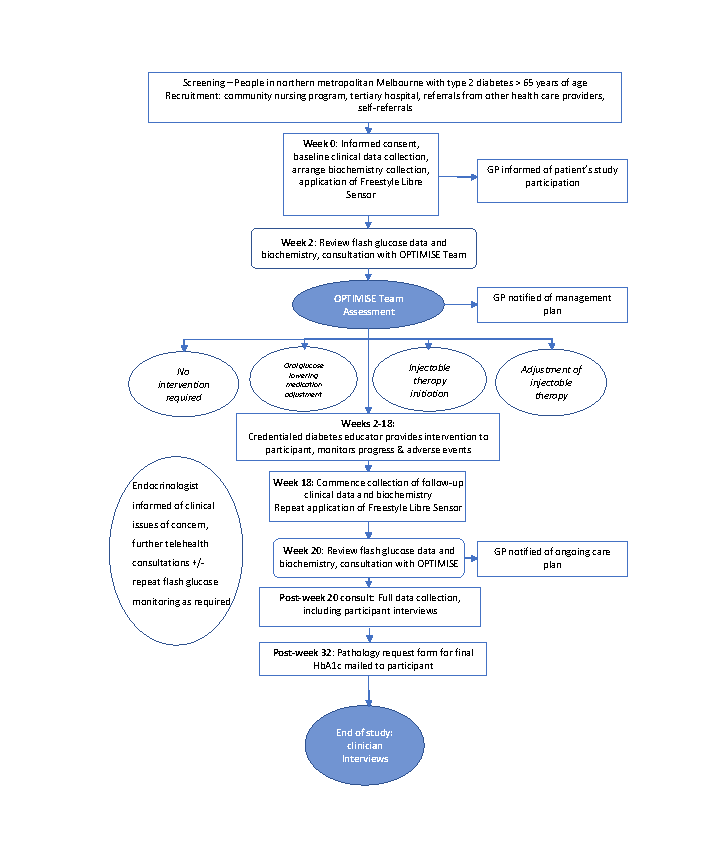

Supplement: Multimedia Appendix 1 [file resprot_v8i6e13986_app1.png]

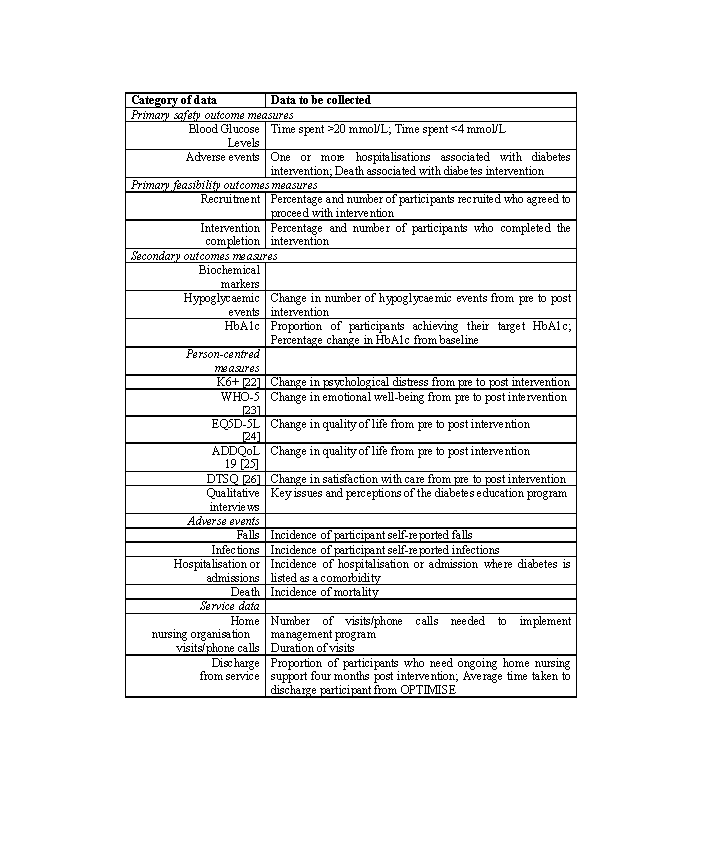

Supplement: Multimedia Appendix 2 [file resprot_v8i6e13986_app2.png]
